# Criminal charge history, handgun purchasing, and demographic characteristics of legal handgun purchasers in California

**DOI:** 10.1186/s40621-021-00301-5

**Published:** 2021-02-08

**Authors:** Veronica A. Pear, Mona A. Wright, Aaron B. Shev, Garen J. Wintemute, Rose M. C. Kagawa

**Affiliations:** Department of Emergency Medicine, University of California Davis School of Medicine, Sacramento, CA USA

**Keywords:** Violence, Gun violence, Crime, Epidemiology

## Abstract

**Background:**

The prevalence and characteristics of handgun purchasers’ criminal charge histories have never been described for a large population of firearm owners, but such information is critical to understanding risk factors for subsequent violence in this population. We sought to characterize legal handgun purchasers in California and compare this group to the state population, to quantify the proportion with a criminal charge history at purchase, and to identify modifiable factors associated with of having such a history.

**Methods:**

This cross-sectional study of all 79,927 legal handgun purchasers aged 21–49 years in California in 2001 used log-linear generalized additive models to identify factors associated with having a criminal charge history at purchase. Subjects are from a longitudinal study of incident criminal activity among handgun purchasers.

**Results:**

The majority (91.03%) of purchasers were male; whites were overrepresented and Hispanics were underrepresented relative to their population size. At the time of purchase, 16.68%  had a criminal charge history and 10.71% had a criminal conviction. Among men with such a history, 31.28% had been charged with a violent crime and 16.54% had been charged with a firearm-related crime. The strongest factor associated with having a criminal charge history was redeeming a pawned handgun (prevalence ratio: 1.82; 95% confidence interval: 1.71, 1.93).

**Conclusions:**

Despite California’s stringent firearm purchase laws, more than 1-in-6 handgun purchasers had a criminal charge history at purchase. This proportion may be higher in states with less restrictive firearm purchasing eligibility criteria.

**Supplementary Information:**

The online version contains supplementary material available at 10.1186/s40621-021-00301-5.

## Background

Firearm violence, both self- and other-directed, is a major threat to public health and was responsible for nearly 40,000 deaths in 2018 alone (Centers for Disease Control and Prevention, National Center for Injury Prevention and Control 2020). Firearm purchasers with a criminal history, including arrests or convictions for misdemeanor offenses, have been identified as being at particularly high risk for committing firearm violence (Wintemute et al. 2001; Wintemute et al. 1998a; Wright and Wintemute 2010; Kagawa et al. 2020). Nevertheless, individuals with extensive histories of criminal charges are legally permitted to purchase firearms as long as they have not been convicted of a prohibiting offense, such as a felony or domestic violence misdemeanor.

The prevalence of legal firearm purchasers who passed a background check and have non-prohibiting criminal charge histories at the time of purchase is not known; neither are the distributions of crime types nor the factors associated with having such a history. Some non-prohibiting criminal charges are associated with an increased risk for future violence. For example, firearm purchasers with a prior DUI conviction have 3.3 times the risk of being charged with a subsequent violent crime compared to firearm purchasers with no history of criminal charges (Wintemute et al. 1998a; Kagawa et al. 2020; Wintemute et al. 2018). Certain purchasing characteristics may also be important. For example, purchasers of assault-type handguns with a criminal charge history have 1.7 times the risk of subsequent arrest for an offense involving firearms or violence than purchasers without a criminal charge history (Wintemute et al. 1998b). Finally, a history of alcohol or drug related offenses may indicate an underlying substance use disorder, which is associated with an increased risk for self-harm (Conner et al. 2019; Anglemyer et al. 2014), including with a firearm (Branas et al. 2016).

Prior research in this area has predominantly comprised surveys among firearm owners, such as the National Firearm Survey and the California Safety and Wellbeing Survey (Azrael et al. 2017; Kravitz-Wirtz et al. 2019). That work is limited by its reliance on self-report and a lack of information on criminal history at the time of firearm acquisition.

This study advances research in the field by using administrative data on demographic features, handguns purchased, and criminal charges to characterize legal purchasers of handguns in California and to identify factors associated with having a criminal charge history at the time of purchase. Our aims are: 1) to characterize the population of legal handgun purchasers in California and compare them to the adult population of the state, 2) to determine the prevalence of criminal charges and convictions among purchasers at the time of purchase, and 3) to determine what modifiable characteristics are associated with having a history of any criminal charges or of specific charge types known to be risk factors for violence at the time of purchase among legal handgun purchasers. To do this, we use baseline data from a large, long-term cohort study that evaluated the association between conviction for driving under the influence and perpetration of violence among handgun purchasers (Kagawa et al. 2020). To our knowledge, this comprehensive dataset of legal handgun purchasers linked to criminal history records is unique.

## Methods

### Definition of terms

We use “purchasers” to refer to all individuals in the Dealer Record of Sale (DROS) data, the state archive of all legal firearm transfers. A transfer is any event resulting in a change in ownership of a firearm; the majority of transfers are sales by licensed retailers, but they also include pawn redemptions, private party transfers, and more.

We use “criminal charge history” to indicate that a person has been charged with a criminal offense. This includes arrests with or without convictions as well as criminal charges accrued through the legal process and is thought to be a better indicator of criminal behavior than convictions alone (Maltz 1984), particularly for violent crimes (Blumstein 1982).

### Study design and participants

This cross-sectional study describes the demographic characteristics of all handgun purchasers in California in 2001 and compares that population to the state population aged 21 years and older (the legal age to purchase a handgun). All handgun purchasers in 2001 were identified using DROS data. Since 1931, firearm dealers have been required to report all handgun transfers to the California Department of Justice (CA DOJ), and since 1991, private party transfers have been required to go through a licensed dealer. As a result, DROS has records for nearly all legal handgun transactions for the state as of 1991. Data for the population of California were gathered from the American Community Survey for 2001 and have been weighted to be representative of the state.

In addition to comparing handgun purchasers to the state’s population, we quantified the proportion of handgun purchasers who had a criminal charge history at the time of purchasing a handgun in 2001 and identified modifiable risk factors for having such a history. In keeping with previous research, we excluded individuals aged 50 years and older for this portion of the analysis, as this population was more likely to have problematic paper-based criminal history records (which are challenging to obtain and abstract) and was considered to be at relatively low risk of engaging in subsequent criminal activities (Loeber and Farrington 2014).

### Measures

Criminal records for the cohort members were extracted from the Criminal History Information System (CHIS) and provided by CA DOJ. As has been described in a previous publication, DROS records were linked to CHIS using a combination of deterministic and probabilistic linking techniques in order to identify cohort members’ criminal charge histories (Kagawa et al. 2020). A series of decision-making rules were applied to links that matched with a lesser degree of certainty and, following this, a team of researchers manually reviewed the remaining links.

Our primary outcome measure was having any criminal charge history at the time of purchasing a handgun. For those with more than one transaction in 2001, criminal charge history was evaluated at the time of the first transaction. We did not limit criminal history to convictions in the primary analysis because these data are known to be incomplete (Bureau of Justice Statistics 2011), but we repeated the analyses using only convictions in a secondary analysis. We also examined specific offenses that have been associated with particularly high risk of future criminal activity. These included all violent offenses—defined following Federal Bureau of Investigation’s (FBI) offense coding guidelines as well as by applying the World Health Organization (WHO) definition of violence—and 3 subcategories of violent offenses: firearm violence, violent offenses included in the FBI’s Crime Index (homicide, rape, aggravated assault, robbery), and sexual or intimate partner violence (IPV) (U.S. Department of Justice, Federal Bureau of Investigation 2004; U.S. Department of Justice, Federal Bureau of Investigation 2018; Violence Prevention Alliance 2019). We also examined alcohol offenses, drug offenses, and the number of previous arrests in secondary analyses.

Independent variables included demographic characteristics, attributes of the handguns purchased in the first transaction in 2001, and the number of legal handguns purchased since 1985 (the earliest data available). Demographic characteristics included age, self-identified race/ethnicity (white, Black, Hispanic, Asian, Native American, and other), sex, county urbanicity, and census tract socioeconomic status (SES). Age, race/ethnicity, sex, and residential county information came from DROS. County urbanicity was determined with the 2003 Rural Urban Continuum Codes, collapsed to metropolitan (codes 1–3) and nonmetropolitan (codes 4–9) (U.S. Department of Agriculture 2004). SES was measured using a principal component analysis on rank-transformed residential census tract variables for education, income, poverty, and unemployment in 2001 (U.S. Census Bureau 2016; Geolytics Estimates Premium [DVD-ROM] 2013).

Characteristics of handguns purchased in 2001 include the number purchased in the subjects’ first transaction and, for each handgun, caliber (classified as small, medium, and large), category (semi-automatic pistol, revolver, or other), and retailer-type-by-transaction-type (non-pawn firearm dealer [e.g. gun shop] sale, private party transfer, or other; and pawn shop sale, redemption, private party transfer, or other). Pawn shops were identified by license type “02,” which is embedded in their Federal Firearm License numbers. To minimize misclassification, we also classified retailers as pawn shops if key words were included in the store name (e.g., “pawn,” “loan”) or if they had 10+ pawn redemptions in 2001. The number of handguns purchased at the index sale in 2001 and the number purchased prior to 2001 were categorized as 1, 2, and 3 or more. This information was obtained from DROS. Previous handgun purchases were identified with data from the Automated Firearms System (AFS) (1985–1995) and DROS (1996–2000). To characterize the purchasing history for cohort members, individuals’ records for 1996–2000 were linked using DROS’ Person ID variable and, for earlier AFS records, using a combination of name, date of birth, city, zip code, county and, where helpful, one of 3 ID types: military, California ID, or driver’s license (Kagawa et al. 2020).

### Statistical analysis

Measures of central tendency, percentages, and corresponding statistics of variability were used to describe the study population. Adjusted log-linear general additive models (GAM) were used to estimate associations between population characteristics and having a criminal charge history at the time of purchase. In the adjusted model examining associations with the number of arrests at the time of purchase, we used a linear GAM. We found the association between age and having a criminal history to be nonlinear, so we modeled age with a cubic spline. We adjusted standard errors to account for multiple observations per person (for those who purchased more than 1 handgun in their first transaction in 2001) and for multiple testing by controlling the false discovery rate (FDR) (Benjamini and Yekutieli 2005). The FDR approach was chosen for its focus on finding the most important effects in a large number of tests. All models included the aforementioned handgun and purchase characteristics and controlled for demographic confounders (age, race/ethnicity, sex, urbanicity, and SES). Observations missing any outcome or covariate data were dropped only in the multivariate analyses (n transactions = 4025 [4.95%]; n people = 3830 [4.81%]). Excluded individuals were more likely than included individuals to live in non-metropolitan counties (14.86 and 3.80%, respectively), but other differences were fairly minor (Supplemental Table 1). Analyses were done with R (version 3.5.3) and StataSE (version 14.2.431).

## Results

There were 110,729 handgun purchasers aged 21 years and older in California in 2001, and 79,927 (72.18%) of these purchasers were between the ages of 21 and 49. Table 1 presents demographic information on these two populations along with the statewide population of residents aged 21 years and older. A much higher percentage of handgun purchasers than of the statewide population were male (91.56 and 48.61%, respectively). Whites and people living in non-metropolitan counties were also overrepresented among handgun purchasers relative to the state population, and Hispanics were underrepresented.

**Table 1:** Demographic characteristics of all California handgun purchasers and state residents in 2001^a^

	Handgun purchasers^b^	Handgun purchasers < 50 years old^c^	State population, 21 + ^d^
Total population	110,729	79,927	22,716,353
Median Age (IQR)	40 (30–51)	34 (27–42)	43 (32–56)
Male (%)	91.56	91.03	48.61
Race/ethnicity (%)			
White	73.37	68.85	51.20
Black	4.90	5.40	5.77
Hispanic	12.68	15.35	27.42
Asian	6.96	8.03	11.85
Native American	0.58	0.60	0.55
Other	1.51	1.77	3.21
Non-metropolitan county (%)	5.20	4.34	2.48^e^

^a^Limited to people aged 21 and older on or after the 10-day waiting period

^b^56 missing race/ethnicity; 1 missing county assignment

^c^29 missing race/ethnicity; 1 missing county assignment

^d^State population is based on data from the American Community Survey for California in 2001, weighted to be representative

^e^Based on data from the 2000 decennial census

Among handgun purchasers between the ages of 21 and 49, 16.68% had a criminal charge history at the time of their first purchase in 2001, including 17.52% of men and 8.19% of women. Table 2 presents demographic details and criminal charge information for those with a criminal charge history, stratified by sex. Men and women with a criminal charge history had roughly the same median age but had distinct racial distributions; a much higher proportion of women than men with a criminal charge history were Black (23.21 and 8.78%, respectively). Of those with a criminal charge history, most had been charged with non-firearm, non-violent offenses (86.53%), followed by violent offenses (31.99%), and alcohol-related offenses (19.34%). The type of criminal charges also differed notably by sex, with men having higher proportions of firearm-, violence-, and alcohol-related charges, and women having higher proportions of drug and non-violent, non-firearm charges. The median number of years between the last criminal charge and the purchase date was 6.89 for men and 5.75 for women.

**Table 2:** Characteristics of California handgun purchasers with a history of criminal charges in 2001, limited to people aged 21 to 49

	Men *N* = 12,706^a^	Women *N* = 586^a^
**Demographics**		
Age (median, IQR)	34 (27–41)	35 (28–41)
Race/ethnicity		
White (%)	7975 (62.77)	331 (56.48)
Black (%)	1115 (8.78)	136 (23.21)
Hispanic (%)	2611 (20.55)	83 (14.16)
Asian (%)	683 (5.38)	23 (3.92)
Native American (%)	95 (0.75)	5 (0.85)
Other (%)	222 (1.75)	8 (1.37)
Unknown (%)	5 (0.04)	–
**Criminal charge history**		
Number of arrests (median, IQR)	2 (1–3)	2 (1–3)
Years since last charge (median, IQR)	6.89 (2.92–12.32)	5.75 (2.55–10.49)
Criminal charge type^b^		
Firearm related (%)^c^	2102 (16.54)	32 (5.46)
Violence (%)	4101 (31.28)	151 (25.77)
Firearm violence (%)	462 (3.64)	12 (2.05)
Crime Index Violence (%)	2140 (16.84)	81 (13.82)
IPV or sexual violence (%)	948 (7.46)	25 (4.27)
Alcohol related (%)	2498 (19.66)	73 (12.46)
Drug related (%)	2408 (18.95)	146 (24.91)
Non-violent, non-firearm (%)	10,975 (86.38)	526 (89.76)

^a^Of all handgun purchasers aged 21–49 years (N men = 72,522; N women = 7156), 17.52% of men and 8.19% of women had a criminal charge at purchase. Criminal history could not be determined for 249 of the 79,927 handgun purchasers aged 21–49, so they were excluded from the denominators

^b^Categories are not mutually exclusive

^c^Firearm related charges include charges related to violence (e.g., assault), trafficking, and possession violations

We found that 10.71% of the cohort had a criminal conviction at the time of purchase. Information about these individuals and their convictions is presented in Supplemental Table 2. Demographic features of those with a conviction and those with a criminal charge were largely similar. As expected, when including only convictions, a lesser proportion of individuals had records involving violence or drugs (13.74 and 6.31%, respectively); convictions for many of these offenses prohibit the purchase of firearms in California.

Handgun and purchase characteristics were largely similar between purchasers with and without a criminal charge history (Table 3). The vast majority (98.53%) of purchasers bought a single handgun and slightly more than half were first-time purchasers. The most notable differences were with regard to retailer and transaction type: those with a criminal charge history had a higher prevalence of redeeming handguns from a pawn shop than those without a criminal history (8.88% vs. 3.32%, respectively). Those without a criminal history were more likely to have a non-pawn shop “other” transaction type, which includes curio/relic, loan, and non-roster peace officer transactions.

**Table 3:** Handgun and purchase characteristics by criminal charge history

	No criminal charge history *N* = 66,386 people (67,773 handguns)	Criminal charge history *N* = 13,292 people (13,611 handguns)
**Index handguns**	N (%)	N (%)
N purchased		
1	65,435 (98.57)	13,071 (98.34)
2	745 (1.12)	172 (1.29)
3+	206 (0.31)	49 (0.37)
Retailer & transaction type^a,b^		
Non-pawn sale	42,830 (63.20)	8533 (62.69)
Non-pawn private party	11,130 (16.42)	2103 (15.45)
Non-pawn other	7147 (10.55)	863 (6.34)
Pawn sale	2794 (4.12)	626 (4.60)
Pawn private party	758 (1.12)	129 (0.95)
Pawn redemption	2249 (3.32)	1209 (8.88)
Pawn other	467 (0.69)	86 (0.63)
Unknown	398 (0.59)	62 (0.46)
Caliber^a^		
Small	7308 (10.78)	1529 (11.23)
Medium	24,955 (36.82)	5210 (38.28)
Large	35,400 (52.23)	6854 (50.36)
Unknown	110 (0.16)	18 (0.13)
Category^a^		
Semi-automatic pistol	51,578 (76.10)	10,341 (75.98)
Revolver	15,679 (23.13)	3133 (23.02)
Other	489 (0.72)	134 (0.98)
Unknown	27 (0.04)	3 (0.02)
**Previous handguns**		
N purchased		
0	35,447 (53.40)	6794 (51.11)
1	9925 (14.95)	2100 (15.80)
2	5696 (8.58)	1298 (9.77)
3+	15,318 (23.07)	3100 (23.32)

^a^N (%) is for number of handguns, not number of people

^b^“Other” includes curio/relic, loan, and non-roster peace officer transactions

Controlling for demographic characteristics, retailer-by-transaction type was the strongest, most consistent characteristic associated with prevalence of criminal charge history among purchasers (Table 4). Pawn redemptions (vs. non-pawn sales) were associated with a 71% greater prevalence of having been charged with firearm violence (prevalence ratio [PR]: 1.71, 95% CI: 1.19, 2.46) and a 129% greater prevalence of having been charged with sexual violence or IPV (PR: 2.29, 95% CI: 1.79, 2.92), with the PRs for other offense types of interest falling within this range. Conversely, non-pawn “other” transactions were associated with a lower prevalence of having a criminal charge history, with PRs ranging from 0.63 for sexual violence or IPV (95% CI: 0.46, 0.86) to 0.42 for firearm violence (95% CI: 0.24, 0.73). The associations between other handgun and purchase characteristics and having a criminal charge history varied by charge type. Variables associated with having an alcohol or drug offense and having more than 1 criminal arrest were similar to those for other types of charges (Supplemental Table 3).

**Table 4:** Adjusted prevalence ratios for having a criminal charge history at the time of purchasing a handgun^a^ (*N* = 75,856 purchasers; 77,359 firearms)

	Any charge	Violence	Firearm violence	Crime Index violence	Sexual violence or IPV
**Index Firearm**					
N purchased					
1	1.00 (Ref.)	1.00 (Ref.)	1.00 (Ref.)	1.00 (Ref.)	1.00 (Ref.)
2	0.93 (0.84, 1.04)	0.84 (0.68, 1.05)	0.84 (0.41, 1.71)	0.73 (0.52, 1.02)	**0.55 (0.32, 0.94)**
3+	1.01 (0.88, 1.16)	**0.65 (0.47, 0.89)**	1.44 (0.72, 2.87)	**0.53 (0.32, 0.88)**	0.79 (0.44, 1.42)
Retailer & transaction type^b^					
Non-pawn sale	1.00 (Ref.)	1.00 (Ref.)	1.00 (Ref.)	1.00 (Ref.)	1.00 (Ref.)
Non-pawn private party	0.95 (0.90, 1.00)	0.93 (0.85, 1.03)	0.75 (0.54, 1.05)	0.91 (0.79, 1.05)	0.88 (0.70, 1.10)
Non-pawn other	**0.63 (0.58, 0.68)**	**0.60 (0.52, 0.70)**	**0.42 (0.24, 0.73)**	**0.54 (0.44, 0.68)**	**0.63 (0.46, 0.86)**
Pawn sale	1.04 (0.96, 1.13)	1.10 (0.94, 1.28)	0.76 (0.43, 1.36)	**1.24 (1.01, 1.53)**	1.22 (0.87, 1.71)
Pawn private party	0.84 (0.70, 1.01)	0.89 (0.64, 1.25)	0.72 (0.22, 2.36)	1.04 (0.67, 1.63)	0.57 (0.22, 1.45)
Pawn redemption	**1.82 (1.71, 1.93)**	**1.81 (1.60, 2.04)**	**1.71 (1.19, 2.46)**	**1.74 (1.46, 2.07)**	**2.29 (1.79, 2.92)**
Pawn other	0.89 (0.72, 1.12)	0.96 (0.64, 1.43)	1.20 (0.36, 4.01)	1.15 (0.68, 1.96)	1.52 (0.76, 3.03)
Caliber					
Small	1.00 (Ref.)	1.00 (Ref.)	1.00 (Ref.)	1.00 (Ref.)	1.00 (Ref.)
Medium	**0.92 (0.87, 0.98)**	0.93 (0.83, 1.04)	1.14 (0.75, 1.73)	0.89 (0.76, 1.05)	1.00 (0.77, 1.30)
Large	**0.90 (0.85, 0.95)**	**0.88 (0.79, 0.99)**	1.12 (0.74, 1.68)	**0.84 (0.72, 0.99)**	0.88 (0.68, 1.13)
Handgun Category					
Semi-auto pistol	1.00 (Ref.)	1.00 (Ref.)	1.00 (Ref.)	1.00 (Ref.)	1.00 (Ref.)
Revolver	**1.10 (1.06, 1.15)**	**1.14 (1.05, 1.24)**	0.96 (0.71, 1.28)	1.11 (0.98, 1.25)	**1.28 (1.07, 1.53)**
Other	**1.24 (1.04, 1.48)**	**1.54 (1.13, 2.09)**	1.70 (0.63, 4.57)	1.41 (0.89, 2.23)	1.12 (0.49, 2.56)
**Previous firearms**					
N purchased					
0	1.00 (Ref.)	1.00 (Ref.)	1.00 (Ref.)	1.00 (Ref.)	1.00 (Ref.)
1	1.01 (0.96, 1.06)	1.05 (0.95, 1.15)	1.34 (0.96, 1.87)	1.01 (0.87, 1.16)	0.92 (0.74, 1.14)
2	**1.07 (1.01, 1.14)**	1.01 (0.90, 1.14)	**1.62 (1.11, 2.35)**	1.09 (0.93, 1.29)	0.89 (0.68, 1.16)
3+	1.00 (0.95, 1.05)	0.97 (0.89, 1.06)	**1.94 (1.45, 2.59)**	0.99 (0.87, 1.12)	0.89 (0.73, 1.08)

^a^95% confidence intervals are displayed in parentheses. All models additionally controlled for age, race/ethnicity, sex, county urbanicity, and census tract socioeconomic status

^b^“Other” includes curio/relic, loan, and non-roster peace officer transactions

Results from the secondary analyses using convictions rather than charges are presented in Supplemental Table 4. In general, results were very similar to the primary analysis. Retailer-by-transaction type continued to have the most consistent and strongest association with all modeled offense types except for alcohol-related convictions, which were more strongly associated with purchasing handguns other than semiautomatic pistols or revolvers, such as derringers (PR: 2.11, 95% CI: 1.29, 3.45). The small number of convictions for firearm violence, Crime Index violence, and sexual violence or IPV among our cohort prevented us from modeling associations for these conviction types.

## Discussion

Having a criminal history is strongly associated with future criminal activity—including violent and firearm-related offenses—among handgun purchasers in California and likely elsewhere (Wintemute et al. 1998a; Kagawa et al. 2020; Wintemute et al. 2018). It is therefore important to characterize this population and identify correlates of having a criminal history among firearm purchasers. This statewide, cross-sectional study is the first to quantify the proportion of legal handgun purchasers with a criminal charge history at the time of the purchase. In total, there were more than 110,000 legal handgun purchasers in California in 2001. We found that nearly 17% of handgun purchasers between the ages of 21 and 49 had a criminal charge at the time of the purchase, including charges for firearm violence, Crime Index violence, and sexual violence or IPV. The main factor associated with having a criminal charge history was redeeming a handgun at a pawn shop.

Our findings with regard to the demographic profile of handgun purchasers in California are consistent with previous survey-based studies of firearm ownership nationally (Azrael et al. 2017; Parker et al. 2017; Smith and Son 2015). We found, as did these surveys, that handgun owners were disproportionately white and male. We also found that Hispanics were the least likely to purchase a handgun of any racial/ethnic group, relative to their population size. This is in line with the 2017 Pew survey on national firearm ownership, which found that a smaller proportion of Hispanics reported owning firearms than did whites or Blacks (the only other racial groups examined) (Parker et al. 2017).

The large proportion of individuals with a criminal history indicates that legal handgun purchasers are not always law-abiding citizens. However, it is worth noting that the majority (83.32%) of purchasers had no criminal history. It is also possible that legal firearm purchasers are less like to have a criminal history than the general population, given the many convictions that would prohibit an individual from legally purchasing a firearm and the correlation between being charged with prohibiting and non-prohibiting offenses. Unfortunately, the proportion of the general population with a criminal history cannot be estimated with publicly available data.

Violent misdemeanor convictions carry a 10-year firearm ownership prohibition in California, yet nearly a third of arrests (and 14% of convictions) among male handgun purchasers with a criminal history were for violent offenses. This seeming contradiction can be explained, in part, by the fact that we defined violence following FBI and WHO guidelines, which are broader than the set of prohibiting violent misdemeanors. In addition, convictions may have been dismissed and the 10-year prohibition may have expired for some individuals, allowing them to legally purchase a handgun in 2001; 13% of individuals with a criminal charge history were last charged with a violent crime more than 10 years prior to purchase.

The strongest, most consistent factor associated with criminal history was redeeming a handgun at a pawn shop. It is known that certain firearm retailers are more likely to be involved with crime than others: pawnbrokers, in particular, are more likely to agree to a straw purchase and crime guns are more likely to originate from pawn shops than other dealers (Wintemute 2010; Wintemute 2009; Wintemute et al. 2005; Wright et al. 2010). Such retailers likely have a reputation in the community that attracts criminally involved individuals, some of whom will be legally permitted to purchase firearms. However, our findings suggest that the potential risk associated with pawn shop transactions stems primarily from pawn redemptions. It is possible individuals facing criminal charges may pawn a firearm to pay for legal fees, redeeming it when and if the charges are dropped. The potentially cyclical relationship between pawn redemptions and criminal charges has not previously been explored in the literature, but it deserves further investigation.

We were surprised to find that so few characteristics of the handguns themselves were associated with criminal history, as 2 previous studies found caliber size to be associated with future criminal activity (Wright et al. 2010; Koper 2013). However, temporal or geographic differences in our study populations and in the outcome of interest (criminal involvement pre- vs. post-purchase) could explain this discrepancy.

The major limitations of this study have to do with generalizability. First, we limited our population to legal handgun purchasers between the ages of 21 and 49, so findings should not be generalized to the entire gun-purchasing population in the state. Additionally, legal purchasers may not be representative of legal owners, let alone owners generally. Second, our data are from 2001 and prior and may not generalize to the present if the rate of non-prohibiting criminal charges has changed substantially over time. Since 2001, property crimes, a common type of nonviolent misdemeanor, have decreased about 15% in California (California Department of Justice 2020), so the number of individuals legally purchasing a handgun with a criminal record has likely declined somewhat as well. Third, handgun purchasers in California are subject to more prohibitions than those in other states and are therefore less likely to have a criminal history than their counterparts outside of California. For this reason, the prevalence of criminal history is likely higher in other states.

Finally, criminal records are imperfect indicators of criminal activity, lacking both sensitivity (not capturing unsolved or unreported crimes) and specificity (implicating innocent people). These errors are likely differential by race, as previous research suggests that racial differences in arrest and convictions may be due to a combination of the differences in the underlying rate of engaging in criminal behavior and in differential treatment by police, policing institutions, and the criminal justice system (Mauer and King 2007; Tonry 2010; Piquero 2008). We controlled for race as a confounder in our analyses but chose not to display the results for this (and other confounding variables) because the relationship between race and criminal charge history is itself confounded by features of systemic racism, resulting in a biased estimate for these measures (Westreich and Greenland 2013).

## Conclusions

Firearm owners with a criminal charge history are at particularly high risk of perpetrating interpersonal and self-directed firearm violence (Wintemute et al. 2001; Wintemute et al. 1998a; Wright and Wintemute 2010; Kagawa et al. 2020; Anglemyer et al. 2014). This study provides a foundational description of a population of legal handgun purchasers, identifies the prevalence of having a criminal record by offense type, and presents purchase characteristics associated with having been charged with specific offenses that have been linked to increased risk of violence. These findings inform our understanding of the diverse and distinct population of individuals that purchase handguns and can be used by public health researchers and policymakers in conjunction with other evidence to inform risk-based approaches to violence prevention.

## Supplementary Information

**Additional file 1.**

## Data Availability

The data that support the findings of this study are available from the California Department of Justice but restrictions apply to the availability of these data, which were used under license for the current study, and so are not publicly available.

## References

[CR1] Anglemyer A, Horvath T, Rutherford G (2014). The accessibility of firearms and risk for suicide and homicide victimization among household members: a systematic review and meta-analysis. Ann Intern Med.

[CR2] Azrael D, Hepburn L, Hemenway D, Miller M (2017). The stock and flow of U.S. firearms: results from the 2015 National Firearms Survey. RSF.

[CR3] Benjamini Y, Yekutieli D (2005). False discovery rate–adjusted multiple confidence intervals for selected parameters. J Am Stat Assoc.

[CR4] Blumstein A (1982). On the racial disproportionality of United states' prison populations. J Crim Law Criminol.

[CR5] Branas CC, Han S, Weibe DJ (2016). Alcohol use and firearm violence. Epidemiol Rev.

[CR6] Bureau of Justice Statistics. . US Department of Justice; 2011.

[CR7] California Department of Justice. Crimes & Clearances. Published 2020. Accessed February 10, 2020. openjustice.doj.ca.gov/exploration/crime-statistics/crimes-clearances.

[CR8] Centers for Disease Control and Prevention, National Center for Injury Prevention and Control. Web-based Injury Statistics Query and Reporting System (WISQARS). Accessed 15, April , 2020. www.cdc.gov/injury/wisqars.

[CR9] Conner A, Azrae D, Miller M (2019). Suicide Case-Fatality Rates in the United States, 2007 to 2014: A Nationwide Population-Based Study. Ann Intern Med.

[CR10] Geolytics Estimates Premium [DVD-ROM] (2013). Geolytics Inc.

[CR11] Kagawa RMC, Stewart S, Wright MA (2020). Association of Prior Convictions for driving under the influence with risk of subsequent arrest for violent crimes among handgun purchasers. JAMA Intern Med.

[CR12] Koper CS (2013). Crime gun risk factors: buyer, seller, firearm, and transaction characteristics associated with gun trafficking and criminal gun use. J Quant Criminol.

[CR13] Kravitz-Wirtz N, Pallin R, Miller M, Azrael D, Wintemute GJ. Firearm ownership and acquisition in California: findings from the 2018 California safety and well-being survey. Inj Prev. 2019;5. 10.1136/injuryprev-2019-043372. 10.1136/injuryprev-2019-043372 31806674

[CR14] Loeber R, Farrington D. Age-crime curve. In: Bruinsma G, Weisburd D, editors. Encyclopedia of Criminology and Criminal Justice: Springer; 2014.

[CR15] Maltz MD (1984). Recidivism.

[CR16] Mauer M, King RS (2007). Uneven justice: state rates of incarceration by race and ethnicity*.* The Sentencing Project.

[CR17] Parker K, Menasce Horowitz J, Igielnik R, Oliphant B, Brown A (2017). The demographics of gun ownership. America’s Complex Relationship with Guns. Pew Reserach Center.

[CR18] Piquero AR (2008). Disproportionate minority contact. Futur Child.

[CR19] Smith T, Son J. Trends in gun ownership in the United States, 1972-2014. Published 2015. Accessed November 8, 2019. https://www.norc.org/PDFs/GSS%20Reports/GSS_Trends%20in%20Gun%20Ownership_US_1972-2014.pdf.

[CR20] Tonry M (2010). The social, psychological, and political causes of racial disparities in the american criminal justice system. Crime Justice.

[CR21] U.S. Census Bureau. American community survey. Accessed 26 October, 2016. http://www.census.gov/programs-surveys/acs/.

[CR22] U.S. Department of Agriculture. Measuring rurality: Rural-urban continuum codes. 2004; Accessed January 17, 2020. https://wayback.archive-it.org/5923/20110913215735/http://www.ers.usda.gov/Briefing/Rurality/RuralUrbCon/

[CR23] U.S. Department of Justice, Federal Bureau of Investigation (2004). Uniform Crime Reporting Handbook.

[CR24] U.S. Department of Justice, Federal Bureau of Investigation (2018). 2019 National Incident-Based Reporting System User Manual.

[CR25] Violence Prevention Alliance. Definition and typology of violence. Accessed 29 January, 2019. https://www.who.int/violenceprevention/approach/definition/en/.

[CR26] Westreich D, Greenland S (2013). The table 2 fallacy: presenting and interpreting confounder and modifier coefficients. Am J Epidemiol.

[CR27] Wintemute GJ (2009). Disproportionate sales of crime guns among licensed handgun retailers in the United States: a case-control study. Inj Prev.

[CR28] Wintemute GJ (2010). Firearm retailers' willingness to participate in an illegal gun purchase. J Urban Health.

[CR29] Wintemute GJ, Cook PJ, Wright MA (2005). Risk factors among handgun retailers for frequent and disproportionate sales of guns used in violent and firearm related crimes. Inj Prev..

[CR30] Wintemute GJ, Drake CM, Beaumont JJ, Wright MA, Parham CA (1998). Prior misdemeanor convictions as a risk factor for later violent and firearm-related criminal activity among authorized purchasers of handguns. JAMA.

[CR31] Wintemute GJ, Wright MA, Castillo-Carniglia A, Shev A, Cerda M (2018). Firearms, alcohol and crime: convictions for driving under the influence (DUI) and other alcohol-related crimes and risk for future criminal activity among authorised purchasers of handguns. Inj Prev..

[CR32] Wintemute GJ, Wright MA, Drake CM, Beaumont JJ (2001). Subsequent criminal activity among violent misdemeanants who seek to purchase handguns: risk factors and effectiveness of denying handgun purchase. JAMA.

[CR33] Wintemute GJ, Wright MA, Parham CA, Drake CM, Beaumont JJ (1998). Criminal activity and assault-type handguns: a study of young aduts. Ann Emerg Med.

[CR34] Wright MA, Wintemute GJ (2010). Felonious or violent criminal activity that prohibits gun ownership among prior purchasers of handguns: incidence and risk factors. J Trauma.

[CR35] Wright MA, Wintemute GJ, Webster DW (2010). Factors affecting a recently purchased handgun's risk for use in crime under circumstances that suggest gun trafficking. J Urban Health.

