# Supplementary material for: Criminal charge history, handgun purchasing, and demographic characteristics of legal handgun purchasers in California
Source: Inj Epidemiol. 2021 Feb 8;8:7. doi: 10.1186/s40621-021-00301-5 (PMC7869452; doi:10.1186/s40621-021-00301-5)
Supplement: Supplementary file 1 — Additional file 1. [file 40621_2021_301_MOESM1_ESM.pdf]

Supplemental Table 1. Characteristics of handgun purchasers included and excluded from multivariable analyses

	**Included**[a]<br>N = 75,856 people<br>(77,359 handguns)	**Excluded**<br>N=3,830 people<br>(4,025 handguns)
**Demographics**		
Age, median (IQR)	34 (27-42)	35 (27-42)
Gender, N (%)		
Male	69,086 (91.08)	3,443 (89.90)
Female	6,770 (8.92)	387 (10.10)
Race/ethnicity, N (%)		
White	51,934 (68.46)	2,938 (76.71)
Black	4,131 (5.45)	167 (4.36)
Hispanic	11,770 (15.52)	423 (11.04)
Asian	6,232 (8.22)	174 (4.54)
Native American	434 (0.57)	45 (1.17)
Other	1,355 (1.79)	54 (1.41)
Unknown	--	29 (0.76)
County Urbanicity, N (%)		
Metropolitan	72,972 (96.20)	3,260 (85.12)
Non-metropolitan	2,884 (3.80)	569 (14.86)
Unknown	--	1 (0.03)
Census Tract SES, median (IQR)	0.21 (-0.58-0.88)	0.32 (-0.42-0.92)
Unknown	--	3,338 (87.15)
**Handguns**		
Index handguns		
N purchased		
1	74,786 (98.59)	3,720 (97.13)
2+	1,070 (1.41)	110 (2.87)
Retailer & transaction type[b]		
Non-pawn sale	49,206 (63.61)	2,157 (53.59)
Non-pawn private party	12,558 (16.23)	675 (16.77)
Non-pawn other	7,657 (9.90)	353 (8.77)
Pawn sale	3,276 (4.23)	144 (3.58)
Pawn private party	844 (1.09)	43 (1.07)
Pawn redemption	3,298 (4.26)	160 (3.98)
Pawn other	520 (0.67)	33 (0.82)
Unknown	--	460 (11.43)
Caliber[b]		
Small	8,324 (10.76)	513 (12.75)
Medium	28,833 (37.27)	1,332 (33.09)
Large	40,202 (51.97)	2,052 (50.98)
Unknown	--	128 (3.18)
Category[b]		
Semi-automatic pistol	59,061 (76.35)	2,858 (71.01)
Revolver	17,739 (22.93)	1,073 (26.66)
Other	559 (0.72)	64 (1.59)
Unknown	--	30 (0.75)
Previous handguns		
N purchased		
0	40,124 (52.89)	2,118 (55.30)
1+	35,732 (47.11)	1,712 (44.70)
**Criminal History**		
Any charges	12,666 (16.70)	626 (16.34)
Violence-related charges	4,035 (5.32)	217 (5.67)
Any convictions	8,131 (10.72)	405 (10.57)
Violence-related convictions	1,103 (1.45)	70 (1.83)

a. 8 people had 1+ transaction included and 1 transaction excluded, and are therefore included in both columns.
b. N (%) is for number of handguns, not number of people.

Supplemental Table 2. Characteristics of Californian handgun purchasers with a history of criminal convictions in 2001, limited to people aged 21 to 49

	**Men** N = 8,192[a]	**Women** N = 344[a]
**Demographics**		
Age (median, IQR)	35 (28-42)	36 (29-41)
Race/ethnicity (%)		
White	5,182 (63.26)	197 (57.27)
Black	715 (8.73)	80 (23.26)
Hispanic	1,677 (20.47)	45 (13.08)
Asian	410 (5.00)	13 (3.78)
Native American	62 (0.76)	3 (0.87)
Other	142 (1.73)	6 (1.74)
Unknown	4 (0.05)	--
**Criminal conviction history**		
Number of convictions (median, IQR)	1 (1-2)	1 (1-2)
Years since last conviction (median, IQR)	7.59 (3.31-13.76)	6.39 (2.99-10.75)
Criminal conviction type (%)[b]		
Firearm related	1,087 (13.27)	9 (2.62)
Violence	1,146 (13.99)	27 (7.85)
Firearm violence	64 (0.78)	0 (0.00)
Crime Index Violence	169 (2.06)	6 (1.74)
IPV or Sexual Violence	71 (0.87)	2 (0.58)
Alcohol related	1,557 (19.01)	51 (14.83)
Drug related	507 (6.19)	32 (9.30)
Non-violent, non-firearm	7,038 (85.91)	325 (94.48)

a. Of all firearm purchasers aged 21-49 years (N men = 72,522; N women = 7,156), 11.30% of men and 4.81% of women had criminal convictions at purchase. Criminal history could not be determined for 249 of the 79,927 handgun purchasers aged 21-49, so they were excluded from the denominators.
b. Categories are not mutually exclusive

Supplemental Table 3. Adjusted prevalence ratios for having a criminal charge history when purchasing a firearm[a] (N=75,856 purchasers; 77,359 firearms)

	**Alcohol offense**	**Drug offense**	**Number of arrests (>0)[b]**
**Index Firearm**			
N purchased			
1	1.00 (Ref.)	1.00 (Ref.)	0.00 (Ref.)
2	1.07 (0.82, 1.42)	0.86 (0.63, 1.15)	**0.11 (0.06, 0.16)**
3+	0.63 (0.38, 1.03)	0.71 (0.45, 1.12)	**-0.88 (-0.92, -0.84)**
Retailer & transaction type[c]			
Non-pawn sale	1.00 (Ref.)	1.00 (Ref.)	0.00 (Ref.)
Non-pawn private party	0.89 (0.78, 1.02)	0.91 (0.79, 1.04)	**-0.11 (-0.13, -0.09)**
Non-pawn other	**0.63 (0.52, 0.76)**	**0.62 (0.51, 0.75)**	**-0.20 (-0.22, -0.18)**
Pawn sale	1.04 (0.83, 1.30)	1.14 (0.92, 1.42)	**0.10 (0.07, 0.13)**
Pawn private party	0.64 (0.37, 1.10)	0.94 (0.60, 1.47)	**-0.10 (-0.17, -0.03)**
Pawn redemption	**2.17 (1.83, 2.58)**	**2.27 (1.92, 2.68)**	**0.31 (0.28, 0.33)**
Pawn other	0.64 (0.33, 1.25)	0.67 (0.35, 1.29)	**0.17 (0.11, 0.23)**
Caliber			
Small	1.00 (Ref.)	1.00 (Ref.)	0.00 (Ref.)
Medium	0.98 (0.83, 1.15)	0.94 (0.80, 1.10)	-0.01 (-0.03, 0.01)
Large	1.01 (0.86, 1.18)	**0.84 (0.72, 0.98)**	**-0.12 (-0.14, -0.10)**
Category			
Semi-automatic	1.00 (Ref.)	1.00 (Ref.)	0.00 (Ref.)
Revolver	**1.15 (1.03, 1.29)**	**1.16 (1.04, 1.30)**	0.01 (-0.00, 0.03)
Other	1.51 (0.97, 2.36)	1.43 (0.92, 2.23)	**0.27 (0.22, 0.33)**
**Previous firearms**			
N purchased			
0	1.00 (Ref.)	1.00 (Ref.)	0.00 (Ref.)
1	0.93 (0.81, 1.06)	0.96 (0.84, 1.10)	-0.01 (-0.03, 0.01)
2	0.91 (0.78, 1.08)	0.93 (0.79, 1.10)	0.00 (-0.02, 0.03)
3+	**0.74 (0.65, 0.83)**	**0.75 (0.66, 0.85)**	**-0.16 (-0.18, -0.15)**

a. 95% confidence intervals are displayed in parentheses. All models additionally controlled for age, race/ethnicity, sex, county urbanicity, and census tract socioeconomic status.
b. Number of arrests was limited to those with at least 1 charge at the time of purchase (N=12,592 purchasers; 12,896 firearms)
c. "Other" includes curio/relic, loan, and non-roster peace officer transactions.

Supplemental Table 4. Adjusted prevalence ratios for having a criminal conviction when purchasing a firearm (N=75,856 purchasers; 77,359 firearms)[a,b]

	**Any Conviction**	**Violent Conviction**	**Alcohol Conviction**	**Drug Conviction**	**Number of Convictions (>0)[c]**
**Index Firearm**					
N purchased					
1	1.00 (Ref.)	1.00 (Ref.)	1.00 (Ref.)	1.00 (Ref.)	1.00 (Ref.)
2	0.86 (0.74, 1.00)	0.89 (0.55, 1.43)	1.10 (0.76, 1.57)	0.62 (0.29, 1.36)	-0.02 (-0.18, 0.14)
3+	0.85 (0.70, 1.05)	0.54 (0.25, 1.17)	0.75 (0.42, 1.34)	0.35 (0.08, 1.51)	**-0.39 (-0.53, -0.24)**
Retailer & transaction type					
Non-pawn sale	1.00 (Ref.)	1.00 (Ref.)	1.00 (Ref.)	1.00 (Ref.)	1.00 (Ref.)
Non-pawn private party	**0.94 (0.87, 1.00)**	0.93 (0.76, 1.14)	0.87 (0.73, 1.03)	0.89 (0.65, 1.23)	0.01 (-0.04, 0.06)
Non-pawn other[d]	**0.63 (0.57, 0.69)**	**0.63 (0.47, 0.85)**	**0.62 (0.49, 0.80)**	**0.56 (0.35, 0.89)**	**-0.10 (-0.17, -0.02)**
Pawn sale	1.06 (0.95, 1.18)	0.88 (0.61, 1.28)	0.91 (0.68, 1.23)	1.31 (0.82, 2.11)	**0.13 (0.04, 0.21)**
Pawn private party	0.88 (0.70, 1.11)	0.76 (0.36, 1.64)	0.55 (0.27, 1.15)	1.70 (0.77, 3.74)	0.02 (-0.18, 0.23)
Pawn redemption	**1.77 (1.63, 1.93)**	**1.60 (1.21, 2.11)**	**2.02 (1.61, 2.53)**	**2.43 (1.66, 3.55)**	**0.20 (0.13, 0.28)**
Pawn other[d]	0.97 (0.74, 1.27)	0.88 (0.38, 2.08)	0.84 (0.41, 1.75)	0.97 (0.29, 3.31)	**-0.15 (-0.29, -0.01)**
Caliber					
Small	1.00 (Ref.)	1.00 (Ref.)	1.00 (Ref.)	1.00 (Ref.)	1.00 (Ref.)
Medium	**0.91 (0.84, 0.99)**	0.87 (0.68, 1.11)	1.01 (0.82, 1.25)	1.00 (0.68, 1.46)	0.01 (-0.06, 0.08)
Large	**0.92 (0.85, 0.99)**	0.89 (0.70, 1.12)	1.07 (0.87, 1.31)	0.97 (0.67, 1.40)	**-0.09 (-0.15, -0.02)**
Category					
Semi-automatic	1.00 (Ref.)	1.00 (Ref.)	1.00 (Ref.)	1.00 (Ref.)	1.00 (Ref.)
Revolver	**1.13 (1.07, 1.20)**	1.14 (0.96, 1.36)	**1.16 (1.01, 1.34)**	1.28 (1.00, 1.65)	-0.01 (-0.06, 0.03)
Other	**1.29 (1.03, 1.63)**	1.74 (0.93, 3.25)	**2.11 (1.29, 3.45)**	1.05 (0.30, 3.69)	-0.02 (-0.18, 0.13)
**Previous firearms**					
N purchased					
0	1.00 (Ref.)	1.00 (Ref.)	1.00 (Ref.)	1.00 (Ref.)	1.00 (Ref.)
1	1.02 (0.95, 1.09)	0.90 (0.73, 1.11)	0.93 (0.78, 1.10)	1.02 (0.76, 1.37)	-0.01 (-0.07, 0.04)
2	1.08 (0.99, 1.17)	0.99 (0.78, 1.27)	0.93 (0.76, 1.15)	0.88 (0.60, 1.28)	0.01 (-0.06, 0.07)
3+	1.06 (1.00, 1.13)	0.84 (0.70, 1.01)	**0.76 (0.65, 0.90)**	**0.62 (0.46, 0.83)**	**-0.07 (-0.11, -0.02)**

a. 95% confidence intervals are displayed in parentheses. All models additionally controlled for age, race/ethnicity, sex, county urbanicity, and census tract socioeconomic status.
b. The number of convictions for firearm violence, sexual violence/IPV, and Crime Index violence were too small for the multivariate analysis to provide meaningful results.
c. Number of convictions is limited to those with at least 1 conviction at the time of purchase (N=8,131 purchasers; 8,314 firearms)
d. "Other" includes curio/relic, loan, and non-roster peace officer transactions.
